# Drainage of Pleural Effusion in the Intensive Care Unit (DOPE‐ICU) Feasibility Trial—Protocol and Statistical Analysis Plan

**DOI:** 10.1111/aas.70233

**Published:** 2026-04-08

**Authors:** Marie Schjødt Worm, Morten Hylander Møller, Niels Henrik Bruun, Anne Craveiro Brøchner, Morten Heiberg Bestle, Bodil Steen Rasmussen, Olav Lilleholt Schjørring

**Affiliations:** ^1^ Department of Anaesthesia and Intensive Care Aalborg University Hospital Aalborg Denmark; ^2^ Department of Clinical Medicine Aalborg University Aalborg Denmark; ^3^ Department of Intensive Care Copenhagen University Hospital—Rigshospitalet Copenhagen Denmark; ^4^ Department of Clinical Medicine, Faculty of Health Sciences University of Copenhagen Copenhagen Denmark; ^5^ Research Data and Biostatistics Aalborg University Aalborg Denmark; ^6^ Department of Anaesthesiology and Intensive Care Lillebælt University Hospital Kolding Denmark; ^7^ Department of Regional Health Research Region of Southern Denmark Odense Denmark; ^8^ Department of Anesthesiology and Intensive Care Copenhagen University Hospital—North Zealand Hillerød Denmark

**Keywords:** critical illness, drainage, intensive care units, pleural effusion, randomised controlled trial, ultrasonography

## Abstract

Pleural effusions are common in adult intensive care unit (ICU) patients with respiratory failure. Ultrasonography‐guided therapeutic pleural drainage is widely used and associated with improved oxygenation and ventilatory parameters, but no randomised clinical trial data exist. This paper outlines the protocol and detailed statistical analysis plan for the Drainage Of Pleural Effusions in the ICU (DOPE‐ICU) feasibility trial. This is an investigator‐initiated, pragmatic, multicentre, open‐label, randomised, parallel‐group feasibility trial. Adults acutely admitted to the ICU with pleural effusion ≥ 2 cm and respiratory failure will be screened for inclusion. We will exclude patients with existing pleural or mediastinal drain, suspected or confirmed haemothorax, pneumothorax, empyema or pleural malignancy, coagulation deficiency or antithrombotic therapy incompatible with drainage, clinical indication for therapeutic pleural drainage and predefined severe hypoxaemic or hypercapnic respiratory failure, expected ICU stay < 24 h, pregnancy, imminent withdrawal of active therapy, or patients previously randomised in the trial. A total of 88 eligible patients will be allocated 1:1 to ultrasonography‐guided therapeutic pleural drainage with small‐bore catheter, with repeated drainage of repeated pleural effusions during ICU stay including readmissions within 90 days versus no therapeutic pleural drainage during ICU stay within 90 days unless pre‐specified escape criteria apply. The primary outcome is a feasibility outcome assessing group separation, i.e., proportion of patients receiving pleural drainage in ICU within 90 days. Secondary clinical outcomes include all‐cause 90‐day mortality, number of patients with one or more serious adverse events within 90 days, and absolute number of days alive without life support and out of hospital, respectively, in 90 days. Additional secondary feasibility and process outcomes will also be investigated. Data will be compared in the intension‐to‐treat population using generalised linear models adjusted for the stratification variable trial site. ClinicalTrials.gov identifier: NCT06709456. The DOPE‐ICU feasibility trial will assess whether randomised allocation to ultrasonography‐guided therapeutic pleural drainage or not in adult ICU patients with respiratory failure and pleural effusion is feasible. This will inform the design of a future large randomised clinical trial evaluating the clinical effects and safety of pleural drainage in this population.

## 1 Introduction

Pleural effusions are common in adult intensive care unit (ICU) patients with reported prevalences between 31% and 81% when assessed ultrasonographically [1, 2, 3, 4, 5]. Common causes of pleural effusion in ICU patients are congestive heart failure, pneumonia, malignancies, hepatic hydrothorax, and nephrotic syndrome [6]. Pleural effusions have been associated with prolonged weaning from mechanical ventilation, increased mortality [7, 8], and longer ICU stay [8]. Pleural drainage is a frequent procedure in the ICU [5], particularly as a therapeutic intervention in ICU patients with respiratory failure [9]. It has been associated with improvements in the partial pressure of arterial oxygen to fraction of inspired oxygen (PaO_2_:FiO_2_) ratio and expiratory lung volumes, whilst carrying a low risk of peri‐procedural complications [10], especially when guided by ultrasonography [10, 11]. However, pleural drainage has also been negatively associated with survival, duration of mechanical ventilation, and ICU and in‐hospital lengths of stay [8]. No published randomised clinical trials have compared pleural drainage with no drainage in ICU patients with respiratory failure and pleural effusions [10]. A smaller Australian randomised trial allocating ICU patients to pleural drainage or no drainage, the Efficacy and Safety Outcomes of Drainage of Intensive Care Pleural Effusions (ESODICE) trial [12], was initiated in 2020 and had enrolled 37 of 52 planned patients by 2023; however, current progress is unclear. Another smaller randomised trial from Egypt allocated 60 mechanically ventilated ICU patients with uncomplicated pleural effusions to either single‐tap thoracentesis or pleural drain insertion and reported a reduction in the duration of mechanical ventilation with drain insertion [13]. In contrast, a recent non‐ICU randomised clinical trial evaluating therapeutic pleural drainage versus no drainage in 136 patients with cardiac failure and pleural effusions found no significant effects on any clinical outcomes [14]. Consequently, the effects of therapeutic ultrasonography‐guided pleural drainage are uncertain, as reflected in current international guidelines [15]. These provide recommendations for pleural drainage on a diagnostic basis, i.e., when malignancy or infection is suspected, but do not offer guidance on the indications for therapeutic pleural drainage [15]. Although expert consensus guidelines specifically addressing therapeutic pleural drainage in the ICU setting exist, they issue no specific recommendations on indications for conducting therapeutic pleural drainage, and their guidance is largely based on expert opinion or low‐certainty evidence, reflecting the limited availability of higher‐quality data [16]. Given the uncertainty regarding the balance of benefits and harms of therapeutic pleural drainage, together with its widespread use in ICUs, the evidence gap highlights the need for large, well‐designed randomised clinical trials. The aim of the Drainage Of Pleural Effusions in the Intensive Care Unit (DOPE‐ICU) feasibility trial is to assess feasibility and provide preliminary safety and outcome data to inform the design of such definitive large‐scale trials. Here we outline the trial rationale, methods, and detailed statistical analysis plan.

## 2 Materials and Methods

### 2.1 Trial Design

The DOPE‐ICU feasibility trial is an investigator‐initiated, pragmatic, multicentre, open‐label, randomised parallel group feasibility trial allocating acutely admitted adult ICU patients with respiratory failure and pleural effusion 1:1 to receive ultrasonography‐guided pleural drainage with a small‐bore catheter or not. Randomisation is centralised and web‐based according to concealed computer‐generated allocation sequence lists with permuted blocks of varying sizes, stratified for trial site.

This protocol and statistical analysis plan has been written according to the Standard Protocol Items: Recommendations for Interventional Trials (SPIRIT) 2013 statement [17], but confers to the updated SPIRIT 2025 statement (see Supporting Information S1 for the SPIRIT checklist) [18].

### 2.2 Approvals, Registration and Ethics

The DOPE‐ICU feasibility trial (current protocol version 2.0 dated 30th July 2025) is approved by the Committee on Health Research Ethics in the North Denmark Region (N‐20240027) and prospectively registered at ClinicalTrials.gov (identifier: NCT06709456). It adheres to the Helsinki Declaration [19], the good clinical practise guidelines [20], and national Danish laws. Consent for enrolment follows Danish regulations on acute trials in temporarily incompetent patients, meaning that consent will be obtained as soon as possible after inclusion from the patient's next of kin and an independent physician, i.e., a trial guardian. Consent from the patient will be obtained if competence is regained.

In Denmark, the Danish Patient Compensation insures all trial participants.

### 2.3 Setting

ICUs in university and non‐university hospitals in Denmark serve as trial sites. All sites are familiar with conducting large‐scale randomised clinical trials and are part of the Collaboration for Research in Intensive Care (CRIC) network. An updated list of participating sites can be found at ClinicalTrials.gov (identifier: NCT06709456).

### 2.4 Inclusion Criteria

All adult patients (age ≥ 18 years), acutely admitted to the ICU, with drainable pleural effusion of ≥ 2 cm in either pleural cavity evaluated by ultrasonography, computed tomography or magnetic resonance imaging, and respiratory failure defined as receiving any oxygen supplementation in an open system, invasive or non‐invasive mechanical ventilation including non‐intermittent CPAP, or as hypercapnic failure with an arterial partial pressure of carbon dioxide (PaCO_2_) > 6.0 kPa and a pH < 7.35, will be screened for eligibility. To ensure timely identification of eligible patients, daily ultrasonographic examinations of pleurae in patients with respiratory failure will be encouraged.

### 2.5 Exclusion Criteria

Patients will be excluded if they fulfil one or more of the following criteria: (1) Mediastinal drain or pleural drain in situ, (2) suspected or confirmed haemothorax, (3) suspected or confirmed pneumothorax, (4) suspected or confirmed pleural empyema, (5) suspected or confirmed pleural malignancy, (6) receives antithrombotic treatment or has a coagulation deficiency incompatible with conducting pleural drainage, and contraindications to reversal of this (clinical assessment), (7) clinically assessed indication for therapeutic drainage and (a) invasive or non‐invasive mechanical ventilation or non‐intermittent mask CPAP with PaO_2_:FiO_2_ ratio ≤ 13.3 kPa, (b) high‐flow humidified oxygen therapy with a flow ≥ 50 L/min and a PaO_2_:FiO_2_ ratio ≤ 13.3 kPa, or (c) persistent respiratory acidosis with a pH < 7.25 and PaCO_2_ > 6.0 kPa in spite of non‐invasive ventilation for > 1 h, (8) Withdrawal from active therapy or brain death is imminent, (9) expected ICU stay < 24 h, (10) pregnancy, (11) under coercive measures, i.e., ongoing involuntary hospital admission or under correctional authorities' jurisdiction, (12) consent not obtainable as per Danish legislation or (13) previously randomised in the DOPE‐ICU feasibility trial.

### 2.6 Screening and Randomisation

All patients fulfilling the inclusion criteria will be screened by local investigators in the centralised web‐based screening system, through the electronic case report form (eCRF). Patients are eligible if they fulfil all inclusion criteria and none of the exclusion criteria.

Inclusion and exclusion of patients will be reported as according to the Consolidated Standards of Reporting Trials (CONSORT) 2025 statement [21].

### 2.7 Interventions

Eligible patients will be randomised to either the intervention group or the control group.

#### 2.7.1 Intervention Group

Patients in the intervention group will receive ultrasonography‐guided pleural drainage with insertion of a small‐bore catheter (typically pig‐tail type catheter, size 6–14 French) in the pleural cavity with pleural effusion of ≥ 2 cm as soon as possible, conducted by radiologists, intensivists, or surgeons, as per local standard. A time interval of up to 24 h from randomisation to intervention is expected to schedule pleural drainage and to allow for sufficient pausing of antithrombotic treatment or correction of coagulation deficiency if required. In case of bilateral pleural effusions ≥ 2 cm at randomisation, drainage of contralateral pleura will be conducted, expected within 24 h from insertion of the first pleural catheter. Inserted pleural catheters remain in situ and open until removed at clinicians' discretion according to local standards. If a new pleural effusion ≥ 2 cm is detected at any time during ICU stay within 90 days from randomisation, including during ICU readmissions, either contralaterally or ipsilaterally after pleural catheter removal, additional pleural drain insertion should be conducted.

#### 2.7.2 Control Group

Patients allocated to the control group will not receive pleural drainage throughout the length of stay in the ICU including readmissions up until 90 days from randomisation, unless pleural drainage is judged clinically indicated, and one or more escape criteria (Table 1) are present as defined by (1) suspected or confirmed haemothorax, (2) suspected or confirmed pneumothorax, (3) suspected or confirmed pleural empyema, (4) suspected or confirmed pleural malignancy, (5) clinically assessed indication for therapeutic drainage and (a) invasive or non‐invasive mechanical ventilation or non‐intermittent mask CPAP with PaO_2_:FiO_2_ ratio ≤ 13.3 kPa, (b) high‐flow humidified oxygen therapy with a flow ≥ 50 L/min and a PaO_2_:FiO_2_ ratio ≤ 13.3 kPa, or (c) persistent respiratory acidosis with a pH < 7.25 and PaCO_2_ > 6.0 kPa in spite of non‐invasive ventilation for > 1 h. If a patient in the control group receives pleural drainage according to an escape criterion, the participant remains in the trial and additional pleural drain insertions within 90 days from randomisation follow the allocated strategy of no drainage. The participant will remain in the intention‐to‐treat population.

**TABLE 1:** Escape criteria for pleural drainage in the control group.

Escape criterion	Definition
Suspected or confirmed haemothorax	By radiographic or ultrasonographic assessment, or by clinical presentation. Assessed by clinicians.
Suspected or confirmed pneumothorax	By radiographic or ultrasonographic assessment, or by clinical presentation. Assessed by clinicians.
Suspected or confirmed pleural infection	By radiographic or ultrasonographic assessment, or by clinical presentation. Assessed by clinicians.
Suspected or confirmed pleural malignancy	Suspected or confirmed pleural lymphoma, pleural metastases or direct pleural invasion of cancer, or malignant mesothelioma. Detected by radiographic assessment, or by anamnesis and clinical presentation Assessed by clinicians.
Invasive or non‐invasive mechanical ventilation with PaO_2_:FiO_2_ ratio ≤ 13.3 kPa	Any mechanical ventilation including mask CPAP with a PaO_2_:FiO_2_ ratio ≤ 13.3 kPa in the most resent ABG conducted at the time of decision to conduct pleural drainage.
High‐flow humidified oxygen therapy with a flow ≥ 50 L/min and PaO_2_:FiO_2_ ratio ≤ 13.3 kPa	In the most resent ABG conducted at the time of decision to conduct pleural drainage.
Respiratory acidosis with a pH < 7.25 and PaCO_2_ > 6.0 kPa in spite of non‐invasive ventilation for > 1 h	In the most resent ABG conducted at the time of decision to conduct pleural drainage.

Abbreviations: ABG: arterial blood gas analysis; CPAP: continuous positive airway pressure; FiO_2_: fraction of inspired oxygen; ICU: intensive care unit; PaCO_2_: arterial partial pressure of carbon dioxide; PaO_2_: arterial partial pressure of oxygen.

#### 2.7.3 Concomitant Interventions

All other interventions are at the discretion of the treating clinicians. In relation to respiratory failure, adherence to updated international clinical guidelines is recommended [22, 23, 24, 25, 26, 27, 28]. Co‐enrolment in other interventional trials will be permitted unless protocol requirements conflict, as determined and approved by the respective trial steering committees.

### 2.8 Withdrawal and Discontinuation of Trial Intervention

If informed consent is not given or retracted at any time (according to Danish regulations), the patient will be withdrawn from the trial. Data registration for a withdrawn patient will continue if consent for this is given. Only the trial participant can demand deletion of already registered data. If so, all data will be deleted, and a new participant will be enrolled to obtain the full sample size.

Clinicians or investigators may deviate from the allocated pleural drainage strategy if the participant experiences intolerable adverse events suspected to be related to the allocated trial intervention or the clinicians in conjunction with the coordinating centre decide it to be in the best interest of the patient. In such cases the patient remains in the trial and the allocated strategy of pleural drainage will be reinstated as soon as it is considered acceptable within 90 days. If a patient after inclusion is subject to involuntary hospitalisation and treatment, the continued strategy of pleural drainage will follow local standards. The collection of data and follow‐up will continue, and the participant will remain in the intention‐to‐treat population.

Participants who are discharged to another ICU will be regarded as discharged from the ICU unless the receiving ICU is an active DOPE‐ICU feasibility trial site. If so, the patient will keep the allocated pleural drainage strategy during ICU admission up until 90 days after randomisation. All patients will be followed up for all outcomes.

### 2.9 Outcome Measures

The primary outcome is a feasibility outcome evaluating group separation of the intervention, defined as the proportion of patients receiving pleural drainage during ICU stay within 90 days from randomisation.

Secondary outcomes are divided into three categories: secondary feasibility outcomes, secondary clinical outcomes, and secondary process outcomes:

#### 2.9.1 Secondary Feasibility Outcomes

- Proportion of included patients with one or more protocol violations, defined as: (1) patients allocated to pleural drainage who do not receive pleural drainage within 24 h from randomisation, (2) patients allocated to pleural drainage with bilateral pleural effusion ≥ 2 cm, who do not receive the second (contralateral) pleural drainage within 24 h from the primary drainage unless pleural drainage has receded < 2 cm, and (3) patients allocated to no pleural drainage who receive pleural drainage at any time during ICU stay within 90 days from randomisation, including ICU readmissions, except when escape criteria for pleural drainage are present as specified (see Table 1).
- Recruitment proportion, defined as the proportion of included patients out of the number of eligible patients, i.e., patients who comply with all inclusion and exclusion criteria over the duration of trial inclusion.
- Proportion of recruited patients with consent withdrawn for the use of data

#### 2.9.2 Secondary Clinical Outcomes

- All‐cause mortality within 90 days from randomisation.
- Proportions of patients with one or more serious adverse events within 90 days from randomisation defined as: (1) new pneumothorax requiring invasive treatment (drainage or surgery), (2) new haemothorax requiring red blood cell transfusion or other haemorrhagic complication related to pleural drainage requiring red blood cell transfusion, (3) new blood stream infection defined as any cultured microorganism from any blood sample except microorganisms clearly specified to be contaminants or likely contaminants by the microbiological department, and (4) new episode of invasive mechanical ventilation defined as endotracheal intubation or re‐intubation after extubation or tracheal decannulation.
- Absolute number of days alive without life support being the use of mechanical ventilation, renal replacement therapy, or circulatory support in the 90‐day period. Mechanical ventilation is defined as invasive or non‐invasive mechanical ventilation or CPAP. Intermittent CPAP is not considered mechanical ventilation. Renal replacement therapy is defined as any form of renal replacement therapy. In patients receiving intermittent renal replacement therapy, days between treatments are included as being with the use of renal replacement therapy. Circulatory support is defined as the use of continuously infused dopamine, norepinephrine, epinephrine, phenylephrine, vasopressin analogues, dobutamine, milrinone or levosimendan.
- Absolute number of days alive and out of hospital in the 90‐day period.

#### 2.9.3 Secondary Process Outcomes

- Proportion of patients with new pleural infection defined as any positive pleural fluid culture of any fungi or bacteria except pre‐defined contaminants being coagulase negative Staphylococci, Enterococci, and *Corynebacterium* species in any pleural fluid sampling conducted after 24 h from randomisation up until 90 days after randomisation.
- PaO_2_:FiO_2_ ratio in the arterial blood gas analysis (ABG) conducted in ICU closest to 24 and 72 h after randomisation. The FiO_2_ in open systems will be estimated from pre‐specified conversion tables (see Table 2)
- pH in the ABG conducted in ICU closest to 24 and 72 h after randomisation.
- PaCO_2_ in the ABG conducted in ICU closest to 24 and 72 h after randomisation.

**TABLE 2:** Conversion tables for calculating FiO_2_ in open systems.

Nasal cannula^a^											
Oxygen flow	0 L/min	1 L/min	2 L/min	3 L/min	4 L/min	5 L/min	6 L/min	8 L/min	10 L/min	12 L/min	14 L/min
FiO_2_	0.21	0.27	0.33	0.37	0.40	0.44	0.48	0.55	0.62	0.64	0.66

High‐flow humidified oxygen^b^																	
Total flow	Percentages represent oxygen concentration in the gas mixture, fractions the corresponding FiO_2_																
	21%	25%	30%	35%	40%	45%	50%	55%	60%	65%	70%	75%	80%	85%	90%	95%	100%
15 L/min	0.21	0.23	0.26	0.29	0.32	0.35	0.38	0.41	0.44	0.47	0.50	0.52	0.55	0.58	0.61	0.64	0.67
20 L/min	0.21	0.24	0.27	0.30	0.33	0.37	0.40	0.43	0.47	0.50	0.53	0.56	0.60	0.63	0.66	0.69	0.73
25 L/min	0.21	0.24	0.28	0.31	0.35	0.39	0.43	0.46	0.50	0.54	0.58	0.61	0.65	0.69	0.73	0.76	0.80
30 L/min	0.21	0.24	0.29	0.33	0.37	0.42	0.46	0.50	0.55	0.59	0.63	0.67	0.72	0.76	0.80	0.85	0.89
35 L/min	0.21	0.24	0.29	0.33	0.38	0.42	0.46	0.51	0.55	0.59	0.64	0.68	0.73	0.77	0.81	0.86	0.90
40 L/min	0.21	0.25	0.29	0.34	0.38	0.43	0.47	0.52	0.56	0.60	0.65	0.69	0.74	0.78	0.83	0.87	0.92
45 L/min	0.21	0.25	0.29	0.34	0.38	0.43	0.47	0.52	0.57	0.61	0.66	0.70	0.75	0.79	0.84	0.88	0.93
> 45 L/min	The FiO_2_ equals the oxygen concentration in the gas mixture																

Hudson mask or similar					
Oxygen flow	6 L/min	8 L/min	10 L/min	15 L/min	30 L/min
FiO_2_	0.45	0.50	0.54	0.59	0.65

Reservoir‐mask (non‐rebreather mask)	
Oxygen flow	≥ 10 L/min
FiO_2_	0.95

*Note:* Used for calculating the PaO_2_:FiO_2_ ratios for the secondary process outcome (i), PaO2:FiO_2_ ratios at 24 and 72 h after randomisation, and in baseline reporting. For flow‐rates or oxygen concentrations not included, the FiO_2_ will be extrapolated from the depicted. Adapted from Waldau et al. [29], Boumphrey et al. [30], Ritchie et al. [31], and Chanques et al. [32].

Abbreviation: FiO_2_: fraction of inspired oxygen.

^a^ Includes oxygen supplementation through T‐piece or speaking valve on tracheostomy.

^b^ Includes any high‐flow oxygen therapy through nasal cannula or high‐flow tracheostomy interface, at flow rates < 15 L/min, use conversion table for conventional nasal cannula above.

### 2.10 Registered Variables

The variables registered at baseline, daily during ICU admission, and at days 2, 4, and 90 are presented in Tables 3, 4, 5, respectively. Complete follow‐up during the 90‐day period for patients transferred to a non‐participating ICU or hospital will be obtained through mail or telephone contact.

**TABLE 3:** Baseline registrations.

Registration	Definition
Age	Calculated from birth date (years).
Sex	Legal sex, as defined by personal identification number in the Danish civil registration system (female/male).
Height	cm.
Weight	Absolute weight (kg).
Date of hospital admission	If transferred from a different hospital, the date of admission to the first hospital applies (ddmmyyyy).
Date and time of ICU admission	Date and time of admission to current ICU (ddmmyyyy, hh:mm).
Pre‐ICU location	(Emergency department or pre‐hospital setting/general ward/operating or recovery room/different ICU).
Surgery	Conducted during current hospitalisation (yes/no).
Surgery type^a^	Of last operation conducted (acute/elective).
Respiratory status	
Estimated pleural effusion size	Right and left side, measured between the parietal and visceral pleura perpendicularly to the chest wall at the largest separation point at randomisation (cm).
Type of respiratory support	At randomisation (Invasive ventilation/NIV including CPAP/oxygen on open systems).
Ventilator settings if invasively ventilated	FiO_2_ (fraction) at the time of the last ABG prior to randomisation, and peak pressure (cm H_2_O), tidal volume (ml) and positive end‐expiratory pressure (cm H_2_O) at the time of randomisation.
Ventilator settings non‐invasively ventilated (including CPAP)	FiO_2_ (fraction) at the time of the last ABG prior to randomisation, and end‐expiratory pressure (cm H_2_O) at randomisation.
If on open system	Type of system (nasal cannula/mask/non‐rebreather mask/T‐piece or speaking valve on tracheal cannula), flow (L/min) and fraction (decimal) of oxygen supplementation at the time of the last ABG prior to randomisation.
PaO_2_	Last ABG before randomisation (kPa).
SaO_2_	Last ABG before randomisation (%).
pH	Last ABG before randomisation.
PaCO_2_	Last ABG before randomisation (kPa).
p‐lactate	Highest plasma lactate measured in 24 h prior to randomisation (mmol/L).
Acute illness^b^	
Pneumonia^a^	As defined by clinicians and noted in the medical records (yes/no).
Side of pneumonia	As estimated by clinicians from radiographic imaging (right‐sided/left‐side/bilaterally).
Multiple trauma	Acute accident with lesions in two anatomical sites or more (yes/no).
Myocardial infarction	Verified by electrocardiographic changes, significant rise in coronary biomarkers, and/or acute PCI or CABG conducted (yes/no).
Cardiac arrest	Clinically diagnosed with initiated cardiopulmonary resuscitation, leading to or occurred during current ICU admission (yes/no).
ARDS	According to the Berlin definition [33] (yes/no).
Sepsis	According to the Sepsis 3 definition [34] (yes/no).
Site(s) of infection^a^	Suspected or confirmed (pulmonary/abdominal/urinary or genital/central nervous system/soft tissue/intravascular/unknown).
SMS‐ICU score [35]	
Systolic blood pressure	Lowest systolic arterial pressure in 24 h prior to randomisation (mm Hg).
Use of vasopressors or inotropes	In 24 h prior to randomisation, includes any continuous infusion of: norepinephrine, epinephrine, phenylephrine, vasopressin analogues, angiotensin, dopamine, dobutamine, levosimendan, or milrinone (yes/no).
Renal replacement therapy	Includes any renal replacement therapy whether chronic or acute, ongoing at the time of randomisation, days between intermittent haemodialysis are considered as with ongoing renal replacement therapy (yes/no).
Chronic co‐morbidity	
Ischaemic heart disease	Previous myocardial infarction, previously conducted PCI or CABG, or previous stable or unstable angina pectoris or use of nitrates indicating this (yes/no).
Chronic heart failure	Chronic LVEF ≤ 40% or diagnosed chronic heart failure with preserved LVEF (yes/no).
Active metastatic cancer	Any metastasis from a malignant non‐haematological neoplasm, which was not considered eradicated at randomisation (yes/no).
Active haematological malignancy	Defined from the World Health Organisation 2017 classification [36], is considered active if the diagnosis resulted in any interventions conducted within the last 6 months prior to randomisation (yes/no).
COPD	Defined as previous spirometry in stable phase diagnostic of COPD [37], or COPD in the anamnesis and daily use of inhaled β_2_‐adrenergic bronchodilators, anticholinergic bronchodilators or glucocorticoids (yes/no).
Chronic dialysis	Includes any renal replacement therapy initiated prior to current hospital admission of any kind, e.g., intermittent haemodialysis or peritoneal dialysis (yes/no).

Abbreviations: ABG: arterial blood gas analysis; ARDS: acute respiratory distress syndrome; CABG: coronary artery bypass grafting; COPD: chronic obstructive pulmonary disease; CPAP: continuous positive airway pressure; FiO_2_: fraction of inspired oxygen; ICU: intensive care unit; LVEF: left ventricular ejection fraction; NIV: non‐invasive ventilation; PaCO_2_: arterial partial pressure of carbon dioxide; PaO_2_: arterial partial pressure of oxygen; PCI: percutaneous coronary intervention; SaO_2_: arterial oxygen saturation of haemoglobin; SMS‐ICU: Simplified Mortality Score for the Intensive Care Unit.

^a^ Only registered if ‘yes’ to previous registration.

^b^ Must have led to or occurred during current hospitalisation.

**TABLE 4:** Daily registrations during ICU admission.

Registration	Definition
Pleural drainage on this day	Includes all types of pleural drainage, e.g., pig‐tail catheter insertion, surgical drainage, or diagnostic pleural tap (yes/no).
Procedure details^a^	Side of pleural drainage (left/right/bilateral), time of drainage (hh:mm), drainage conducted by (radiologist/intensivist/surgeon/other), indication for drainage (pleural effusion ≥ 2 cm in intervention group/haemothorax/pneumothorax/pleural infection/pleural malignancy/invasive or non‐invasive ventilation and PaO_2_:FiO_2_ ratio ≤ 13.3 kPa/high‐flow oxygen therapy with flow ≥ 50 L/min and PaO_2_:FiO_2_ ratio ≤ 13.3 kPa/respiratory acidosis despite non‐invasive ventilation for > 1 h/other).
Pleural catheter or drain in situ on this day	Includes catheters or drains inserted or removed on this day (yes/no).
Drainage details^a^	Side of drain (left/right/bilateral), 24‐h fluid output (mL), pleural catheter removed (yes/no) with time (hh:mm) of removal.
PaO_2_	In the ABG conducted closest to 20:00 in ICU (kPa).
SaO_2_	In the ABG conducted closest to 20:00 in ICU (%).
Type of oxygen supplementation	At the time of the ABG conducted closest to 20:00 in ICU (closed system/open system/none).
FiO_2_ if closed system	At the time of the ABG conducted closest to 20:00 in ICU (fraction).
Oxygen supplementation if open system	Type of system (nasal cannula/mask/non‐rebreather mask/T‐piece or speaking valve on tracheal cannula), flow (L/min) and fraction (decimal) of oxygen supplementation, at the time of the ABG conducted closest to 20:00 in ICU.
PaCO_2_	Highest PaCO_2_ measured on this day in ICU (kPa).
pH	In the ABG with the highest PaCO_2_ on this day in ICU.
Mechanical ventilation	Any use of NIV, continuous mask CPAP, or invasive mechanical ventilation (yes/no). Intermittent CPAP does not count as mechanical ventilation.
PEEP, EPAP or CPAP^b^	Setting closest to 20:00 (cm H_2_O).
Tidal volume^c^	Measurement closest to 20:00 (ml).
Peak pressure^c^	Measurement closest to 20:00 (cm H_2_O).
Mechanical ventilation in prone position	Any proning of the patient due to respiratory failure (yes/no).
Continuous infusion of vasopressors or inotropes	Includes norepinephrine, epinephrine, phenylephrine, vasopressin analogues, angiotensin, dopamine, dobutamine, levosimendan, or milrinone (yes/no).
Renal replacement therapy	Any continuous or intermittent renal replacement therapy (yes/no). Days between any intermittently used renal replacement therapy counts as ‘yes’.
Use of any diuretics	(yes/no).
Diuretic type and dose^a^	Cummulated dose of furosemide (mg), thiazides (mg), spironolactone (mg) other—describe (mg).
Serious adverse events	
Pneumothorax	Any pneumothorax on this day requiring invasive treatment with drainage or surgery (yes/no).
Haemothorax	Any haemothorax on this day, or other haemorrhagic complication to pleural drainage, requiring red blood cell transfusion (yes/no).
New episode of invasive mechanical ventilation	Endotracheal intubation or re‐intubation after extubation or tracheal decannulation on this day (yes/no). Tracheotomy on already intubated patient, endotracheal tube exchange does not count as a new episode of invasive mechanical ventilation. Intubation in relation to surgical procedures does not count as a new episode of invasive mechanical ventilation, unless the patient is transferred to the ICU whilst still intubated after surgery.

*Note:* Daily variables are registered from 06:00 to 06:00 whilst admitted to the ICU for up to 90 days, starting at the time of randomisation and including readmissions.

Abbreviations: ABG: arterial blood gas analysis; CPAP: continuous positive airway pressure; EPAP: expiratory positive airway pressure; FiO_2_: fraction of inspired oxygen; ICU: intensive care unit; NIV: non‐invasive ventilation; PaCO_2_: arterial partial pressure of carbon dioxide; PaO_2_: arterial partial pressure of oxygen; PEEP: positive end‐expiratory pressure; SaO_2_: arterial oxygen saturation of haemoglobin.

^a^ Only registered if ‘yes’ to previous registration.

^b^ Only registered if mechanically ventilated on this day.

^c^ Only registered if ‘invasive’ mechanical ventilation closest to 20:00.

**TABLE 5:** Registrations on day 2 and 4, and at 90‐day follow‐up.

On day 2 and 4	
Registration	Definition
PaO_2_	In the ABG conducted in ICU closest to 24 h and 72 h after randomisation, respectively (kPa).
SaO_2_	In the ABG conducted in ICU closest to 24 h and 72 h after randomisation, respectively (kPa).
Type of oxygen supplementation	At the time of the ABG conducted in ICU closest to 24 h and 72 h after randomisation, respectively (closed system/open system/none).
FiO_2_ if closed system	At the time of the ABG conducted in ICU closest to 24 h and 72 h after randomisation, respectively (fraction).
Oxygen supplementation if open system	Type of system (nasal cannula/mask/non‐rebreather mask/T‐piece or speaking valve on tracheal cannula), flow (L/min) and fraction (decimal) of oxygen supplementation, at the time of the ABG conducted in ICU closest to 24 h and 72 h after randomisation, respectively.
PaCO_2_	In the ABG conducted in ICU closest to 24 h and 72 h after randomisation, respectively (kPa).
pH	In the ABG conducted in ICU closest to 24 h and 72 h after randomisation, respectively (kPa).

At 90‐day follow‐up	
Registration	Definition
Pneumothorax outside the ICU	Any pneumothorax requiring invasive treatment with drainage or surgery within 90 days from randomisation (yes/no).
Haemothorax outside the ICU	Any haemothorax, or other haemorrhagic complication to pleural drainage, requiring red blood cell transfusion within 90 days from randomisation (yes/no).
Blood stream infection	Any blood culture conducted within 90 days from randomisation, with growth of any microorganism except microorganisms clearly specified to be contaminants or likely contaminants by the microbiological department (yes/no). If yes, species and genus of microorganism(s) cultured (descriptive).
Pleural infection	Any pleural fluid culture of any fungi or bacteria, except coagulase negative Staphylococci, Enterococci, and *Corynebacterium* species, in any pleural fluid sampling conducted after 24 h after within 90 days from randomisation (yes/no). If yes, species and genus of microorganism(s) cultured (descriptive).
Pleural drainage outside the ICU	Within 90 days from randomisation, includes all types of pleural drainage, e.g., pig‐tail catheter insertion, surgical drainage, or diagnostic pleural tap (yes/no).
New invasive mechanical ventilation in non‐participating ICU	Endotracheal intubation, or re‐intubation after extubation or tracheal decannulation within 90 days from randomisation conducted in or with transfer to non‐participating ICU (yes/no). Tracheotomy on already intubated patient and endotracheal tube exchange does not count as a new episode of invasive mechanical ventilation. Intubation in relation to surgical procedures does not count as a new episode of invasive mechanical ventilation, unless the patient is transferred to the ICU whilst still intubated after surgery.
Days with mechanical ventilation outside participating ICU	Days from 06:00 to 06:00 with any use of NIV, continuous mask CPAP, or invasive mechanical ventilation in non‐participating ICU, on general ward, or after discharge from hospital in 90 days from randomisation (number of days). Intermittent CPAP does not count as mechanical ventilation. Nocturnal CPAP due to sleep apnoea or obesity‐hypoventilation syndrome does not count as mechanical ventilation.
Days with vasopressors outside participating ICU	Days from 06:00 to 06:00 with any use of norepinephrine, epinephrine, phenylephrine, vasopressin analogues, angiotensin, dopamine, dobutamine, levosimendan, or milrinone in non‐participating ICU in 90 days from randomisation (number of days).
Days with renal replacement therapy outside participating ICU	Days from 06:00 to 06:00 with any continuous or intermittent renal replacement therapy in non‐participating ICU, at general ward or after discharge from hospital in 90 days from randomisation (number of days). Days between any intermittently used renal replacement therapy counts as with renal replacement therapy.
Discharge from hospital	Within 90 days from randomisation (yes/no).
Date of hospital discharge^a^	(ddmmyyyy).
Readmission to hospital^a^	Within 90 days from randomisation (yes/no).
Dates of readmissions^b^	Number of readmissions (*n*) and dates of readmission and discharge for each hospital readmission within 90 days from randomisation (ddmmyyyy).
Dead	Death by any cause within 90 days from randomisation (yes/no).
Date of death^c^	(ddmmyyyy).

Abbreviations: ABG: arterial blood gas analysis; FiO_2_: fraction of inspired oxygen; ICU: intensive care unit; PaCO_2_: arterial partial pressure of carbon dioxide; PaO_2_: arterial partial pressure of oxygen; SaO_2_: arterial oxygen saturation of haemoglobin.

^a^ Only registered in ‘yes’ to ‘discharge from hospital’.

^b^ Only registered if ‘yes’ to ‘readmission to hospital’.

^c^ Only registered if ‘yes’ to ‘dead’.

### 2.11 Blinding

Trial interventions will not be blinded for investigators, clinicians and patients, as this is not considered feasible. Mortality assessments will be registry‐based where possible. The statistical analyses of all secondary outcomes will be done first, with the intervention groups masked, i.e., randomly coded as 0 and 1, and without data extraction on the primary outcome, as this would likely reveal trial allocation for most patients. Afterwards, data on the primary outcome will be extracted, and the primary statistical analysis of this will be conducted, also with allocations masked. All supplemental analyses will be conducted without blinding of statisticians.

### 2.12 Data Registration and Monitoring

All data will be entered into a central web‐based, password protected, encrypted eCRF constructed in Research Electronic Data Capture (REDCap) hosted in the North Denmark Region [38, 39]. All patients will undergo centralised day‐to‐day monitoring by the sponsor or delegate following a defined monitoring plan, according to the International Conference on Harmonisation of technical requirements for registration of pharmaceuticals for human use GCP (ICH‐GCP) standards [40].

### 2.13 Safety and Adverse Events

Selected serious adverse events possibly related to the intervention, as defined under secondary clinical outcome (e), will be registered daily during ICU admission in the eCRF. Similar events occurring after discharge from the ICU will be captured in the eCRF in the 90‐day follow‐up form. When a pneumothorax or haemothorax is registered in the eCRF, an automatic e‐mail notification is sent to the sponsor and delegate, who, together with local investigators, will review each event to assess potential causality between the event and the intervention. Other serious adverse events according to ICH‐GCP definition [40], not defined under secondary clinical outcome (e), as well as non‐serious adverse events, will not be systematically recorded, but will be evaluated continuously by primary site investigators and co‐investigators in their daily clinical practise. If an unexpected serious adverse event occurs, i.e., not defined under secondary clinical outcomes (e), which local investigators consider potentially related to the intervention, sponsor or delegate will be contacted as soon as possible, and potential causality will be assessed. As this is a small, low‐risk feasibility trial, with no pre‐planned interim analyses, no formal data monitoring and safety committee will be established, ongoing safety oversight will be conducted by the investigators and sponsor as described.

### 2.14 Statistics

#### 2.14.1 Sample Size Calculation

To detect or reject an expected minimum of 75% relative increase (an absolute increase of 30% points) in patients receiving pleural drainage in the intervention group, and assuming a maximal incidence of pleural drainage in the ICU within 90 days of 40% in the control group, with a type 1 error risk (two‐sided α) of 0.05 and a type 2 error risk (*β*) of 0.20 (equal to a power of 80%), and an added 4 patients due to an expected dropout rate of maximum 5%, a total of 88 patients will be included. The trial is only powered to show relevant differences in the primary feasibility outcome; consequently, results in all secondary outcomes should be considered exploratory only.

#### 2.14.2 General Analytical Principles

All analyses will be conducted in the intention‐to‐treat population being all randomised patients except those where outcome data cannot be obtained due to withdrawal of consent [40, 41, 42]. Given the limited sample size and lack of power to assess clinical outcomes, no interim analyses will be conducted to avoid inflating type I error.

#### 2.14.3 Outcome Analyses

The primary outcome will be analysed using a generalised linear model with a log‐link and Poisson data distribution according to the relation between variance and mean, and a robust variance estimation adjusted for the stratification variable trial site. Significance of the intervention will be assessed based on the *p*‐value from this regression and reported with the readily available risk ratio with 95% confidence interval. A *p*‐value below 0.05 will be considered statistically significant. A similar analysis, however, with an identity‐link and Poisson data distribution instead to produce the risk difference with 95% CI, will be conducted as well. Dichotomous secondary outcomes will be analysed similarly to the primary outcome; however, no *p*‐values will be reported given the lack of power and explorative nature of these outcomes. Continuous and ordinal secondary outcomes will be analysed using a generalised linear model with an identity‐link, Gaussian data distribution and a robust variance estimation, adjusted for the stratification variable trial site to produce a mean difference with 95% CI. A generalised linear model will be applied, regardless of expected non‐normal data distribution of the residuals. Similarly to the other secondary outcomes, no *p*‐values will be reported.

Additional per‐protocol sensitivity analyses will be conducted for all secondary clinical and process outcomes, excluding patients with one or more protocol violations as defined under secondary feasibility outcome (a).

For the secondary clinical outcome (e) proportion of patients with one or more serious adverse events, a supplemental analysis will be conducted in which death from any cause within 90 days will be included as a serious adverse event.

For the secondary clinical outcome (f) number of days alive without life support, a supplemental analysis will be conducted in which patients who die within 90 days will be assigned a value of 0 days alive without life support.

For the secondary process outcomes (i), (j), and (k), supplemental analyses will be conducted investigating differences in the PaO_2_:FiO_2_ ratio, PaCO_2_ and pH, respectively, between individual baseline and 72‐h measurements.

#### 2.14.4 Baseline Parameters and ICU Treatment

Baseline variables and daily ICU treatment variables will be summarised descriptively according to group allocation. Categorical variables will be presented as counts and percentages, whilst continuous variables will be reported as means with standard deviations or medians with interquartile ranges, as appropriate. No statistical comparisons between groups will be performed for baseline characteristics unless specifically requested during peer review.

#### 2.14.5 Missing Data

If less than 5% of data are missing for the primary outcome, a complete‐case analysis will be performed without imputation of missing values. If more than 5% of data are missing, multiple imputation using chained equations will be applied, generating at least 10 imputed datasets under the assumption that data are missing at random [43, 44]. All outcomes and major prognostic baseline variables will be included in the imputation model. Major prognostic baseline variables include: estimated size of the largest pleural effusion (cm), location of pleural effusion ≥ 2 cm (right, left, or bilateral), age, active metastatic cancer, active haematological malignancy, chronic obstructive pulmonary disease, type of admission (medical, elective surgical, or emergency surgical), and SMS‐ICU score. If multiple imputation is applied, the primary trial analysis will be based on the imputed datasets. The unadjusted, non‐imputed (complete‐case) analysis will also be reported. In addition, a best‐worst and worst‐best case sensitivity analysis will be conducted to explore the potential impact of data that may be missing not at random. In the best‐worst‐case scenario, all participants lost to follow‐up in the experimental group will be assumed to have received pleural drainage in the ICU within 90 days, whilst none with missing data in the control group will be assumed to have received pleural drainage. Conversely, in the worst‐best‐case scenario, none of the participants lost to follow‐up in the experimental group will be assumed to have received pleural drainage, whilst all those lost to follow‐up in the control group will be assumed to have received it.

### 2.15 Dissemination

The trial results will be sought published in an international peer‐reviewed medical journal regardless of the results. We will adhere to the CONSORT 2025 statement when reporting our results [21]. All documents, including protocol amendments, will be available on https://www.cric.nu/dope‐icu/. Protocol changes are communicated to relevant parties by email. De‐identified data may be obtained upon reasonable request after publication of the trial results. For the current protocol publication, no data were used to support this.

## 3 Discussion

The effects of therapeutic pleural drainage in ICU patients with respiratory failure remain largely unknown and existing evidence is conflicting. The DOPE‐ICU feasibility trial will allocate ICU patients with pleural effusions to therapeutic pleural drainage versus no pleural drainage, thereby addressing this important issue [10].

The definition of the size of clinically significant pleural effusion and ideal cut‐off for pleural drainage requirement remain unclear. Proposed cut‐off varies from 1.5 to 4.5 cm in existing literature [2, 7]. A cut‐off of ≥ 2 cm as indicative of need for pleural drainage has been suggested specifically in the ICU [45], equal to 400 mL of pleural effusion volume according to the Balik formula [46]. Consequently, we selected this cut‐off, supported by a recent study from our group that confirmed a high prevalence of pleural effusions above this threshold amongst ICU patients (31%), of which 40% were drained [5].

To ensure timely screening and inclusion of patients in this feasibility trial before pleural drainage is conducted, we include patients with mild respiratory failure, defined as receipt of any supplemental oxygen or mechanical ventilation, or the presence of respiratory acidosis; positive effects of pleural drainage have been reported regardless of oxygen supplementation level [10, 45].

### 3.1 Strengths and Limitations

The strengths of the DOPE‐ICU feasibility trial include the pragmatic study design with all other treatments following routine practise ensuring external validity, whilst minimising the potential risk of protocol violations in trials with complex interventions in ICU [47]. Since the effect of therapeutic pleural drainage is unclear [16], clinicians might be biassed to not examine for pleural effusion in patients with mild respiratory failure. It is therefore encouraged to examine daily for pleural effusion in all ICU patients with respiratory failure during trial conduct. The trial is a randomised multicentre trial which increases external validity with centralised randomisation using concealed computer‐generated allocation sequence lists. To minimise the risk of bias, all analyses will be performed in the intention‐to‐treat population and the current protocol and statistical analysis plan will be published prior to inclusion of the last patient and prior to any data analyses [48]. There are several limitations to this trial. Since it is not feasible to conceal the trial intervention to the clinician, patient and investigators, the trial is not blinded, increasing the risk of bias. The primary outcome of this trial is a feasibility outcome and all secondary outcomes, including all clinical outcomes, will be exploratory only due to the limited sample size.

## 4 Conclusions

The DOPE‐ICU feasibility trial will assess whether randomised allocation to either ultrasonography‐guided therapeutic pleural drainage or no drainage in adult ICU patients with respiratory failure and pleural effusion is feasible. The trial represents an important and necessary step towards the design of a definitive large‐scale randomised clinical trial aimed at providing relevant evidence evaluating the clinical effects and safety of therapeutic drainage of pleural effusions in adult ICU patients.

### 4.1 Trial Status

The first patient was included on December 3rd, 2024. The trial is ongoing, recruiting at four active Danish sites. By March 11th, 2026, a total of 76 patients had been randomised. The last patient is expected to be included in April 2026.

## Author Contributions

M.S.W. and O.L.S. drafted the protocol and the manuscript for this paper in close collaboration with M.H.M. and B.S.R. N.H.B., A.C.M., and M.H.B. all made substantial contributions to the manuscript and provided important scientific input. All authors have read and approved the final manuscript. M.S.W., M.H.M., N.H.B., B.S.R., and O.L.S. are members of the DOPE‐ICU feasibility trial Steering Committee with O.L.S. as sponsor and principal investigator of the trial, and M.S.W. as coordinating investigator. M.S.W., M.H.M., A.C.B., and M.H.B. are site investigators.

## Funding

The DOPE‐ICU feasibility trial is funded by ‘Læge Sofus Carl Emil Friis og Hustru Olga Doris Friis' legat’ [FID4401466] (650.000 DKK) and ‘Region Nordjyllands Sundhedsvidenskabelige Forskningsfond’ [2024–0049] (90.000 DKK). The funding sources will have no influence on the trial design, the scientific content of the protocol, the trial conduct, the data collection or data analysis, the interpretation of the results, or the final publication.

## Conflicts of Interest

The authors declare no conflicts of interest.

## Supporting information


**Supporting Information: S1.** SPIRIT 2025 checklist for Drainage Of Pleural Effusion in the Intensive Care Unit (DOPE‐ICU) feasibility trial—protocol and statistical analysis plan.

## Data Availability

The data that support the findings of this study are available from the corresponding author upon reasonable request.
